# Transcriptomic analysis and machine learning modeling identifies novel biomarkers and genetic characteristics of hypertrophic cardiomyopathy

**DOI:** 10.3389/fgene.2025.1596049

**Published:** 2025-06-17

**Authors:** Feng Zhang, Chunrui Li, Lulu Zhang

**Affiliations:** ^1^ Department of Intensive Care Unit, People’s Hospital of Linquan County, Linquan, China; ^2^ Department of Cardiovascular Medicine, People’s Hospital of Linquan County, Linquan, China

**Keywords:** hypertrophic cardiomyopathy, gene expression, RNA sequencing, gene expression omnibus, DNA repair, biomarker, machine learning

## Abstract

**Objective:**

This study aimed to leverage bioinformatics approaches to identify novel biomarkers and characterize the molecular mechanisms underlying hypertrophic cardiomyopathy (HCM).

**Methods:**

Two RNA-sequencing datasets (GSE230585 and GSE249925) were obtained from the Gene Expression Omnibus (GEO) repository. Computational analysis was performed to compare transcriptomic profiles between normal cardiac tissues from healthy donors and myocardial tissues from HCM patients. Functional annotation of differentially expressed genes (DEGs) was performed using Gene Ontology (GO) and Kyoto Encyclopedia of Genes and Genomes (KEGG) enrichment analyses. Immune cell infiltration patterns were quantified via single-sample gene set enrichment analysis (ssGSEA). A predictive model for HCM was developed through systematic evaluation of 113 combinations of 12 machine-learning algorithms, employing 10-fold cross-validation on training datasets and external validation using an independent cohort (GSE180313).

**Results:**

A total of 271 DEGs were identified, primarily enriched in multiple biological pathways. Immune infiltration analysis revealed distinct patterns of immune cell composition. Based on the top differentially expressed genes, a robust 12-gene diagnostic signature (COMP, SFRP4, RASD1, IL1RL1, S100A8, S100A9, ESM1, CA3, MYL1, VGLL2, MCEMP1, and MT1A) was constructed, demonstrating superior performance in both training and testing cohorts.

**Conclusion:**

This study utilized bioinformatics approaches to analyze RNA-sequencing datasets, identifying DEGs and distinct immune infiltration patterns in HCM. These findings enabled the construction of a 12-gene diagnostic signature with robust predictive performance, thereby advancing our understanding of HCM’s molecular biomarkers and pathogenic mechanisms.

## Introduction

Cardiovascular disease remains the leading cause of death worldwide (Hartman et al., 2024). Hypertrophic cardiomyopathy (HCM) is characterized by asymmetric ventricular septal hypertrophy, leading to left ventricular outflow tract obstruction, impaired diastolic filling, and reduced ventricular compliance (Xu et al., 2021). In severe cases, HCM can result in heart failure, myocardial ischemia, and sudden death. Therefore, early detection of HCM is crucial. However, the mechanism underlying cardiac remodeling in HCM remains unclear (Gu et al., 2024).

Several transcription factors, including SP1 and EGR1, exhibit a fetal-like pattern of binding motifs in nucleosome-depleted regions of HCM (Gao et al., 2024). Previous studies have offered the most extensive map of sex-specific variations in the transcriptome, proteome, and phosphoproteome at the time of surgical myectomy for obstructive HCM (Garmany et al., 2024). Therefore, there are nuanced yet biologically significant differences in the multi-omics profiles of HCM. Lu et al. comprehensively profiled the transcriptomic programs of HCM cardiomyocytes and provided insights into the molecular mechanisms underlying disease pathogenesis (Lu et al., 2024). However, it is important to emphasize the heterogeneity of cardiomyocytes in both healthy and diseased states (Gu et al., 2023). Hence, conducting multicenter studies involving samples from diverse ethnic groups and regions is important to unravel the pathological mechanism of HCM and to gain additional insights into the disease.

Comprehensive transcriptomic profiling of HCM samples using RNA sequencing (RNA-seq) has revealed circulating miRNA biomarkers and dysregulated pathways (Liang et al., 2023). A combination of DNA methylation and transcriptomic profiling has also helped identify and develop new therapeutic targets for HCM (Li et al., 2023). Joshua et al. identified region-specific myocardial gene transcription patterns as well as novel genes and pathways associated with HCM (Joshua et al., 2023). Transcriptomic analysis may provide mechanistic insights into unexplained HCM phenotypes and offer specific genes for potential use as HCM biomarkers or targets in future RNA-targeting therapies (James et al., 2021). Therefore, molecular and functional profiling may aid in guiding precise therapies for HCM (Vakrou et al., 2018). The heterogeneity of cardiomyocytes underscores the necessity of multicenter studies, which are critical for deepening our understanding of HCM pathogenesis to inform clinical diagnosis and treatment (Albulushi et al., 2025).

To address these gaps, we analyzed the latest HCM transcriptomic datasets from the Gene Expression Omnibus (GEO) database. Using machine learning algorithms, we identified key genes and evaluated model performance via area under the curve (AUC) analysis.

## Materials and methods

### RNA-seq dataset acquisition and processing

The publicly accessible Gene Expression Omnibus (GEO) database (https://www.ncbi.nlm.nih.gov/geo/) hosts datasets from various disease investigations. RNA-seq transcriptomic data and clinical metadata for HCM patients were obtained from GEO using R (version 4.4.1). The GSE230585, GSE249925 (Garmany et al., 2024), and GSE180313 datasets comprised myocardial tissue samples from HCM patients and healthy donors. Raw data were processed into an expression matrix, background-corrected, and normalized using the limma R package (version 3.60.6). Batch effects were corrected using the “ComBat” function from the sva package (version 3.52.0) (Leek et al., 2012). Differentially expressed genes (DEGs) between HCM and healthy tissues were identified using the limma R package. External validation was performed using the GSE180313 RNA-seq dataset, which included 27 HCM patients and 13 normal controls (Ranjbarvaziri et al., 2021). Parameters for the pheatmap package (version 1.0.12) were configured using its official documentation (https://www.rdocumentation.org/packages/pheatmap/versions/1.0.12/topics/pheatmap).

### DEG identification and analysis

Differentially expressed genes (DEGs) were identified using the limma package, with significant DEGs defined by |log2(fold change)| > 2 (Ritchie et al., 2015). A false discovery rate (FDR) < 0.05 was set as the significance threshold. Volcano plots and heatmaps of DEGs were generated using the ggplot2 and pheatmap R packages. Functional enrichment analysis of DEGs included Gene Ontology (GO), Kyoto Encyclopedia of Genes and Genomes (KEGG), and Gene Set Enrichment Analysis (GSEA). GSEA was performed with 10,000 permutation tests, and results were visualized using the ggplot2 R package. The top 20 DEGs (10 upregulated, 10 downregulated) were selected for machine-learning model development. Statistical significance was defined as both nominal and adjusted P-values <0.05.

### Enrichment analyses and immune cell infiltration

Gene Ontology (GO) analyses, including biological process (BP), cellular component (CC), and molecular function (MF) analyses, Kyoto Encyclopedia of Genes and Genomes (KEGG) analysis, and Disease Ontology Semantic and Enrichment analysis (DOSE) of differentially expressed genes (DEGs) were performed using the R-package clusterProfiler (https://bioconductor.org/packages/clusterProfiler). Differentially expressed genes (DEGs) were subjected to Gene Set Enrichment Analysis (GSEA) using several R packages: ReactomePA, org. Hs.e.g.,.db, clusterProfiler, biomaRt, and enrichplot. We conducted enrichment analyses to identify the potential biological functions and pathways associated with hypertrophic cardiomyopathy (HCM). We determined significantly enriched KEGG pathways using the net enrichment score, gene ratio, and P-value. A gene set was deemed enriched if the nominal P-value <0.05 and the false discovery rate (FDR) < 0.05. We used single - sample gene set enrichment analysis (ssGSEA) to quantify the levels of 23 infiltrating immune cell types in each sample (Cai et al., 2022).

### Machine-learning algorithms

Twelve machine-learning algorithms were selected, including Naive Bayes, XGBoost, Linear Discriminant Analysis (LDA), Ridge, Generalized Boosted Regression Modeling (GBM), Support Vector Machine (SVM), Elastic Net (Enet), StepGLM, Partial Least Squares Regression for Generalized Linear Models (plsRglm), Lasso, Generalized Linear Model Boosting (glmBoost), and Random Forest (RF) (Chen et al., 2024). A systematic exploration of 113 algorithm combinations was performed on the training dataset, integrating variable selection within a 10-fold cross-validation framework. External validation utilized an independent cohort (GSE180313).

The process began with preprocessing the raw data, which involved removing missing values and outliers, followed by applying Z-score normalization to transform each feature so that its mean became 0 and standard deviation 1. This step effectively eliminated the impact of differing feature scales.

Subsequently, the dataset was randomly split into a training set and a test set, with 70% allocated to the training subset and 30% to the test subset. During the model training stage, various machine learning algorithms were employed to assess their performance. These included Elastic Net regression (λ = 0.1), Lasso regression (λ = 0.05), Ridge regression (λ = 1.0), Support Vector Machine (SVM, with C = 1.0 and γ = 0.01), Linear Discriminant Analysis (LDA), Gradient Boosting Machine (GBM, featuring a 0.1 learning rate and 100 trees), Random Forest (RF, containing 200 trees), and XGBoost (XGB, with a 0.01 learning rate and 150 trees). Each model was trained on the training set, and hyperparameters were optimized via cross-validation.

For model evaluation, the area under the receiver (AUC) value of each algorithm was computed using the test set with a threshold set at 0.7 to gauge classification performance. Based on the previously published literature, we determined the model based on the average AUC value of the training set and the test set. The model with the highest AUC value (Qin et al., 2023) and the appropriate number of genes (Chen et al., 2024) was identified as the optimal model. Calibration plots were generated to evaluate the diagnostic model’s predictive consistency and reliability.

### Statistical analysis

All bioinformatics analyses and visualizations were performed using R (version 4.4.1) on macOS. Non-normally distributed variables were compared using the Mann-Whitney U test. Categorical variables were assessed for statistical significance using the chi-square test or Fisher’s exact test. Gene correlations were quantified using Pearson’s correlation coefficient. Receiver Operating Characteristic (ROC) curves were constructed using the pROC package, and corresponding Area Under the Curve (AUC) values were calculated. Enrichment analyses were considered statistically significant when P-values <0.05 or adjusted P-values (q-values) < 0.05. For all other analyses, statistical significance was defined as a two-tailed P-value <0.05.

## Results

### Data processing and batch effect correction

**FIGURE 1:**
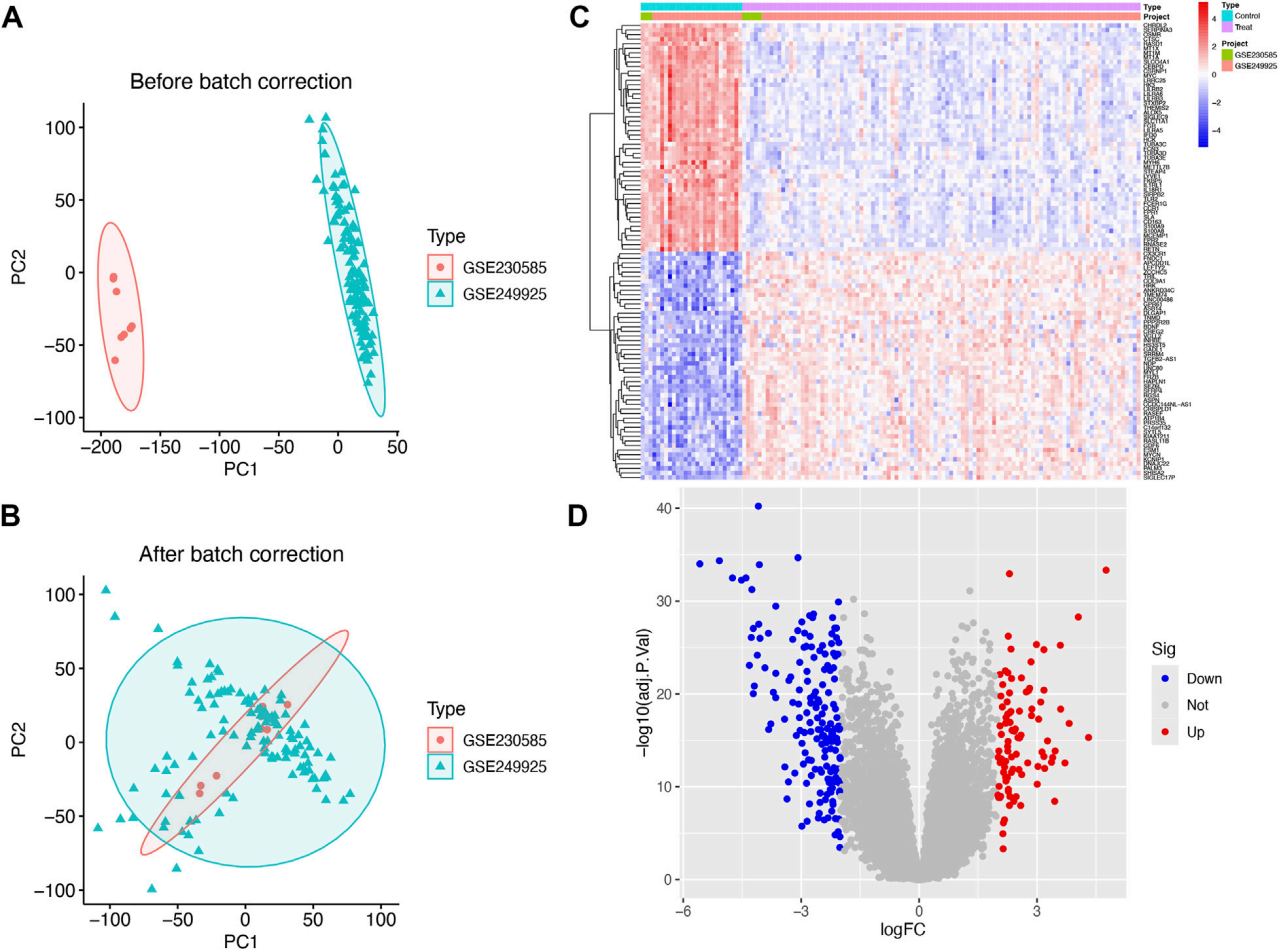
The integration of datasets and differentially expressed genes (DEGs) between heart healthy donors (control) and hypertrophic cardiomyopathy (HCM) patients. **(A)** PCA of two original HCM datasets prior to **(A)** and after **(B)** batch-effect correction. **(C)** Heatmap of DEGs between the control and HCM groups. **(D)** Volcano plot of the DEGs. Significant DEGs (|fold-change| > 2; False discovery rate <0.05) are indicated in red (upregulated) or blue (downregulated).

Transcriptomic datasets from HCM patients and healthy control groups were obtained from the Gene Expression Omnibus (GEO) repository (Table 1). Raw data were preprocessed through batch effect correction, dataset integration, and normalization using established bioinformatics pipelines. Following these systematic workflows, the final processed dataset was generated, as illustrated in Figure 1A and B (Figure 1). Supplementary Table S1 lists all genes included in the study, while Supplementary Table S2 presents the number of differentially expressed genes (DEGs) identified under varying threshold conditions.

**TABLE 1:** Basic information of GEO datasets used in the study.

GSE series	Disease^a^	Samples	Source types	Platform	Group
GSE230585	HCM	5 HCM and 3 normal controls	Cardiac tissue	GPL21697	Discovery cohort
GSE249925	HCM	97 HCM and 23 normal controls	Cardiac tissue	GPL24676	Discovery cohort
GSE180313	HCM	27 HCM and 13 normal controls	Cardiac tissue	GPL24676	Validation cohort

^a^ HCM, hypertrophic cardiomyopathy.

### DEGs between control and HCM samples

The GSE230585 dataset included myocardial tissue samples from 5 HCM patients and three healthy donors, while GSE249925 contained samples from 97 HCM patients and 23 healthy donors. A total of 271 differentially expressed genes (DEGs) were identified between HCM and normal myocardial tissues (Supplementary Table S3). Expression levels of these DEGs were visualized in a heatmap (Figure 1C), with 95 genes upregulated and 176 downregulated. A volcano plot (Figure 1D) was generated to visualize DEGs by fold change and statistical significance, highlighting genes with the most substantial expression differences.

### DEG enrichment analysis

**FIGURE 2:**
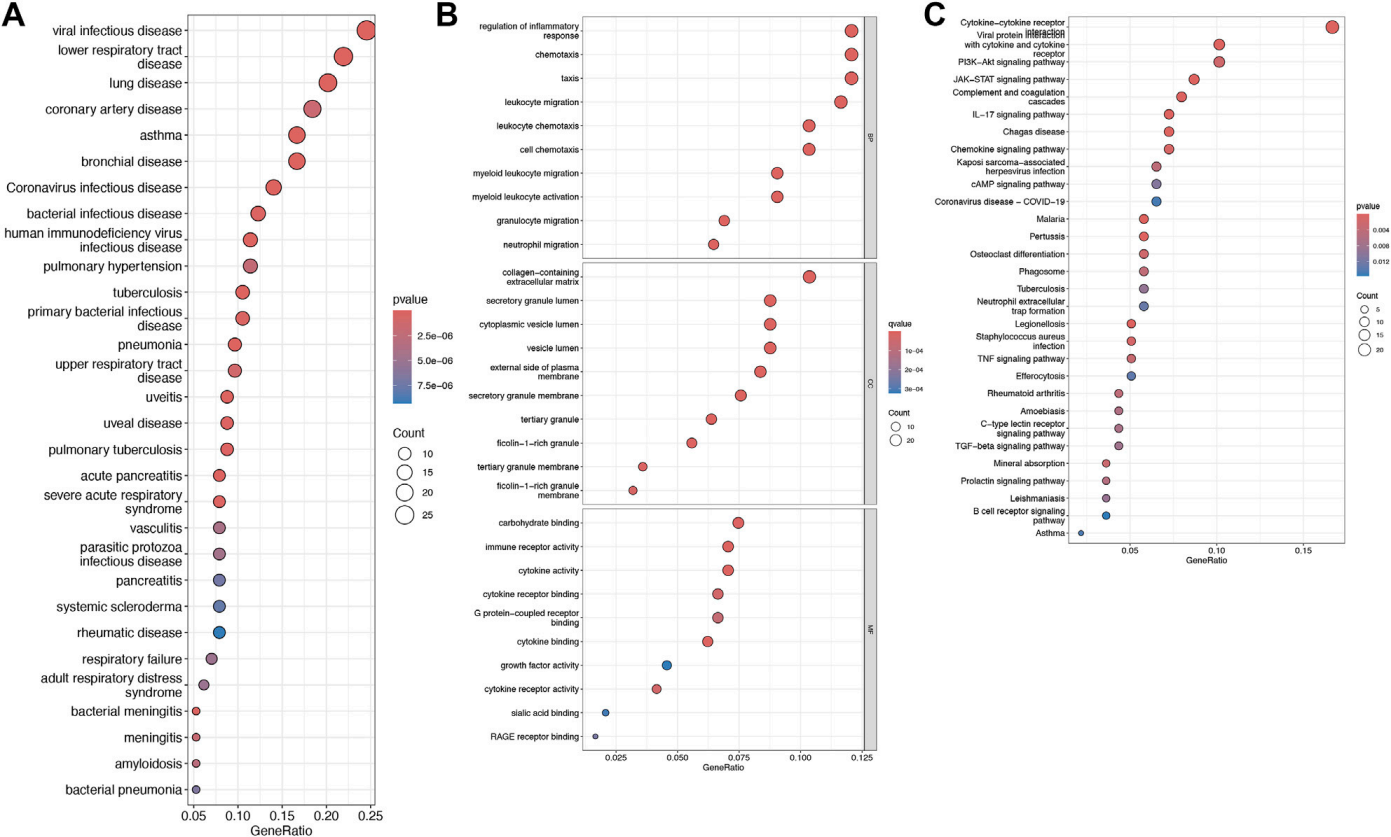
Disease Ontology, Gene ontology (GO), and Kyoto Encyclopedia of Genes and Genomes (KEGG) pathway enrichment analysis of the differentially expressed genes (DEGs). **(A)** Bubble plot showing the DO enrichment results. **(B)** Bubble plot showing that DEGs between the control and HCM groups were enriched in several biological processes (BP), cell components (CC), and molecular functions (MF). **(C)** Bubble chart illustrating the DEG-enriched KEGG pathways. The terms are shown on the y-axis and their enrichment scores are shown on the x-axis. The size of each bubble positively correlates with the number of associated genes, with a higher pathway enrichment P-value intensifying the pink hue of the bubble.

Disease Ontology Semantic and Enrichment analysis additionally showed that DEGs were significantly associated with viral infectious diseases, lower respiratory tract disease, and lung disease (Figure 2A). Several biological functions were identified through GO enrichment analysis of the DEGs. In the BP analysis, DEGs were highly enriched in the regulation of inflammatory responses and chemotaxis. In the CC analysis, the DEGs were highly enriched in the collagen-containing extracellular matrix, secretory granule lumen, and cytoplasmic vesicle lumen. Moreover, MF analysis indicated significant enrichment of DEGs in carbohydrate binding, immune receptor activity, and cytokine activity (Figure 2B). KEGG pathway enrichment analysis of the DEGs revealed significant enrichment, including cytokine-cytokine receptor interaction with cytokine and cytokine-receptor, and PI3K-Akt signaling pathways (Figure 2C).

### Immune cell infiltration analysis

**FIGURE 3:**
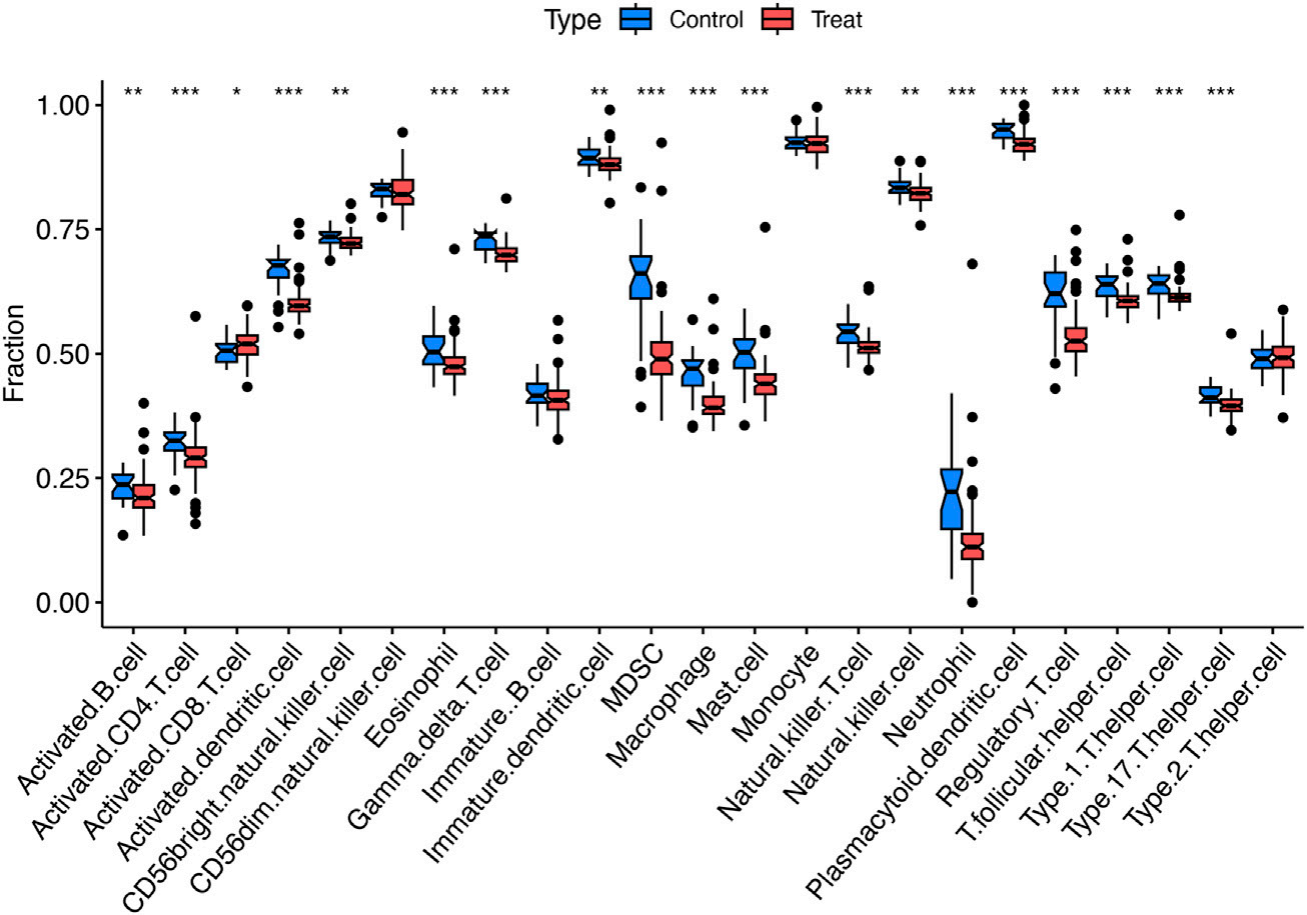
Immunological characteristics. Boxplots comparing immune cell abundances between HCM vs. controls. ***P < 0.001, **P < 0.01, *P < 0.05.

Single-sample gene set enrichment analysis (ssGSEA) was utilized to characterize the composition of immune cell subsets within these cohorts. The boxplot in Figure 3 reveals that the HCM cohort exhibits a higher proportion of activated CD8^+^ T cells, while the abundance of activated B cells, CD4^+^ T cells, activated dendritic cells, and other cell types is lower (Figure 3).

### GSEA of the top five DEGs

**FIGURE 4:**
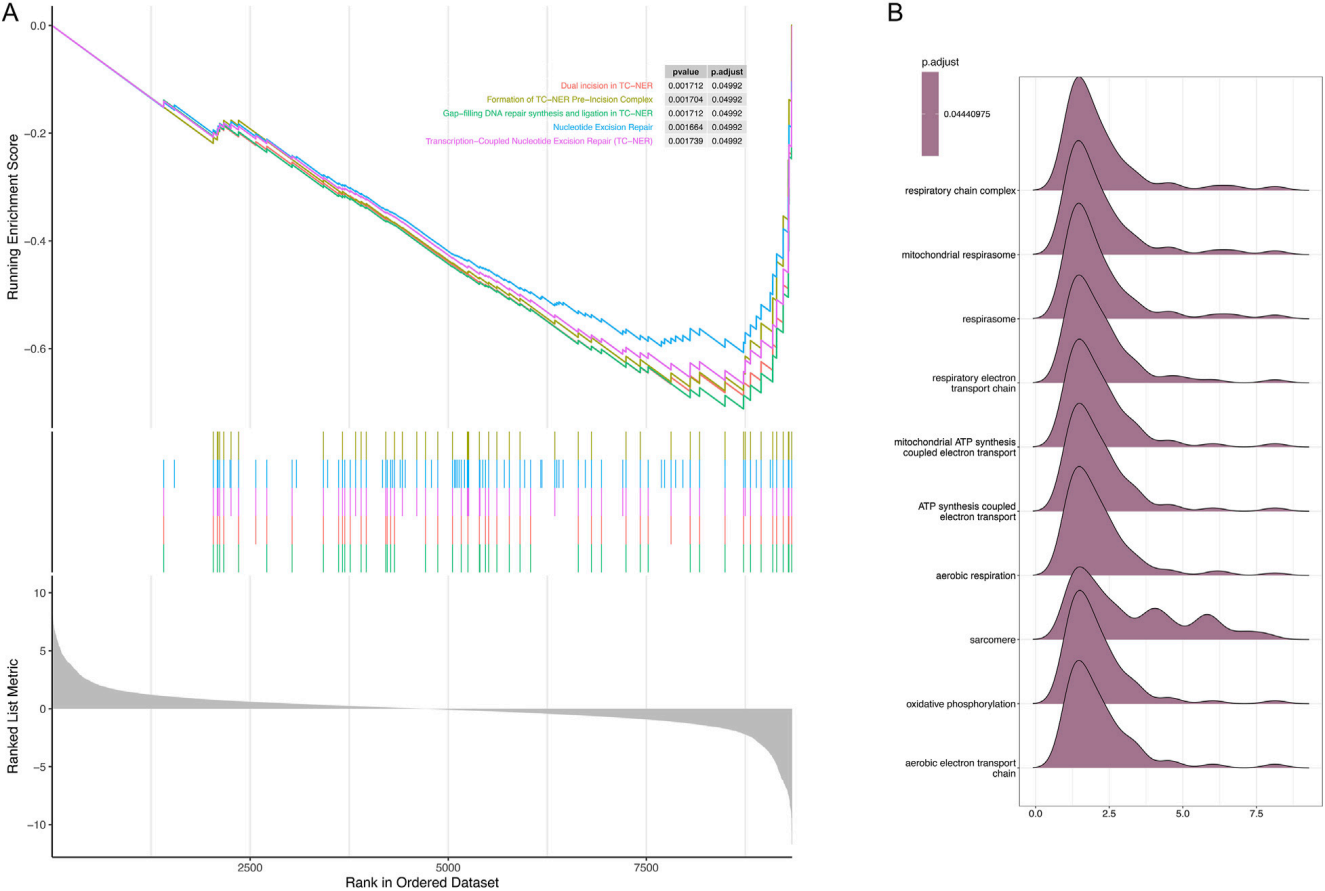
Gene set enrichment analysis (GSEA) of the top five differentially expressed genes (DEGs) between the control and hypertrophic cardiomyopathy (HCM) samples. **(A)** Significant GSEA sets of DEGs. **(B)** Ridge plots showing enrichment of different gene sets.

Further analysis was performed to characterize the specific signaling pathways enriched with differentially expressed genes (DEGs) and the molecular mechanisms underlying their roles in hypertrophic cardiomyopathy (HCM). Enriched pathways included dual incision in transcription-coupled nucleotide excision repair (TC-NER), formation of the TC-NER pre-incision complex, gap-filling DNA repair synthesis, and ligation processes in both TC-NER and nucleotide excision repair (all P < 0.05; Figure 4A). The enrichment fraction curve indicated that these genes exhibited a left-tailed peak, signifying their enrichment at the top of the ranked gene list (adjusted P-value = 0.044; Figure 4B).

### Identification of the most top 10 regulated genes with diagnostic value and developing a diagnostic model for HCM via machine learning

**FIGURE 5:**
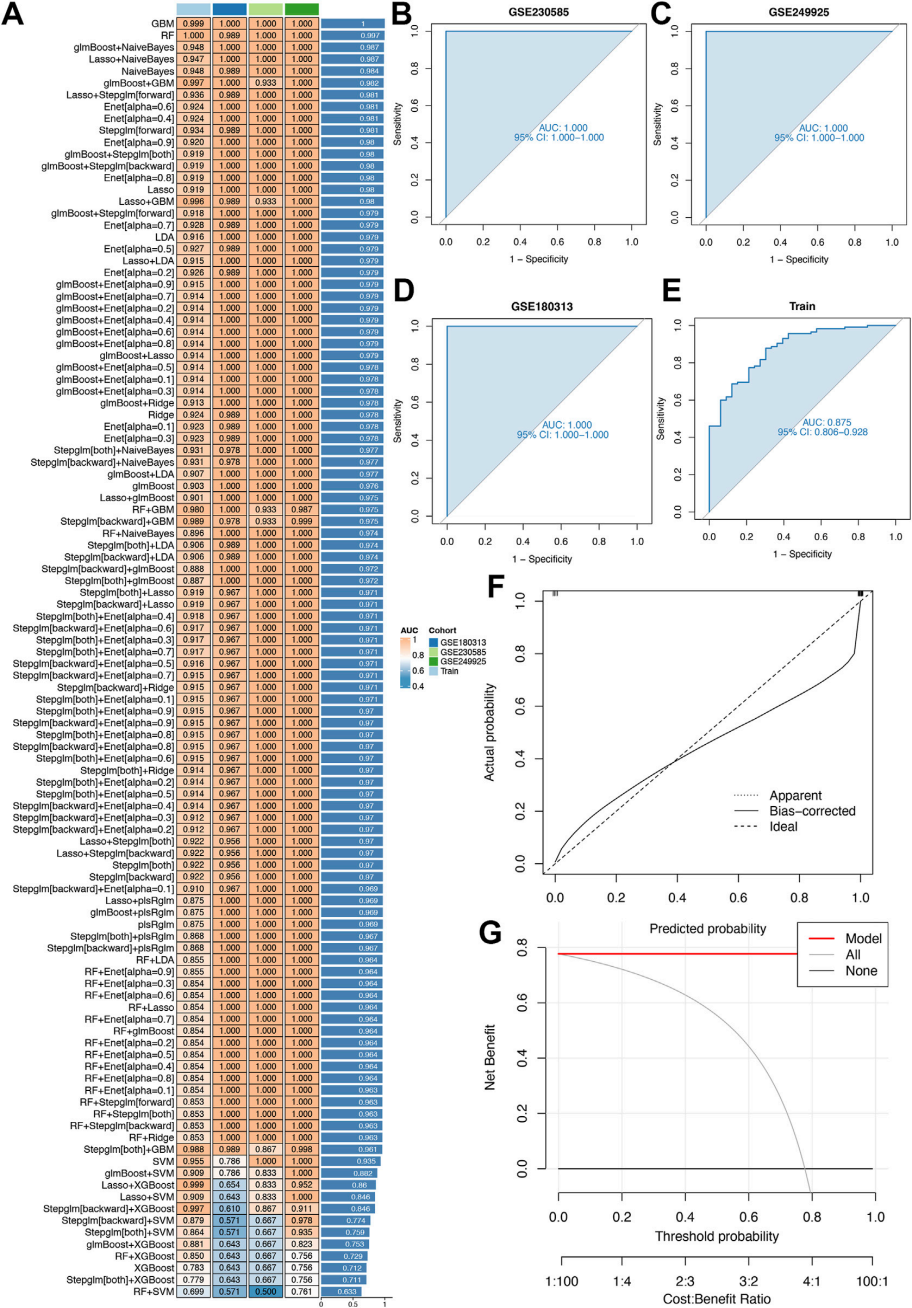
Diagnostic performance of our model. **(A)** 113 machine learning algorithm combinations evaluated via 10-fold cross-validation. **(B,C)** The receiver-operating characteristic (ROC) curves for two distinct validation cohorts (GSE230585 and GSE249925), assessing algorithmic accuracy in these datasets. **(D)** The ROC curves for an external independent validation cohort (GSE180313), testing the model’s generalizability beyond primary datasets. **(E)** The ROC curves for the training cohort, evaluating in-sample model fit. **(F)** The calibration curve assesses the alignment between predicted and observed outcomes to ensure accuracy. **(G)** Clinical decision-curve analysis evaluates the net clinical benefit at different threshold probabilities for the Lasso and Stepglm[both] algorithm within the model. The x-axis represents the threshold probability (0–1) and the y-axis represents the net benefit.

Twelve machine learning algorithms were integrated within a 10-fold cross-validation framework to develop a robust diagnostic model using the top 20 differentially expressed genes (DEGs). The model development process was performed on the training dataset and independently validated using an external cohort (GSE180313), as outlined in Figures 5A–E. The optimal model, exhibiting superior predictive performance, was constructed by integrating the Lasso and Stepglm[both] algorithms. This hybrid approach identified 12 critical genes (COMP, SFRP4, RASD1, IL1RL1, S100A8, S100A9, ESM1, CA3, MYL1, VGLL2, MCEMP1, and MT1A), with the StepGLM algorithm refining the prediction framework for reliability. Calibration plots (Figures 5F,G) demonstrated strong agreement between predicted probabilities and observed clinical outcomes across all cohorts, characterized by near-ideal diagonal distributions. This close correspondence indicates excellent model calibration and consistent performance in estimating disease probabilities.

## Discussion

Hypertrophic cardiomyopathy (HCM) is a complex genetic disorder characterized by myocardial hypertrophy and fibrosis, yet its molecular pathogenesis remains not fully understood (Lee et al., 2025). This study identified 271 differentially expressed genes (DEGs) between HCM patients and healthy controls, highlighting dysregulated pathways, including extracellular matrix (ECM) organization, immune response, and calcium signaling. These findings align with recent studies that have demonstrated the critical role of ECM remodeling in HCM progression (Viola et al., 2023). Additionally, enrichment of immune-related pathways, including cytokine-cytokine receptor interaction, underscores the emerging role of inflammation in HCM pathogenesis, corroborating a study by Pay et al., which identified pan-immune inflammatory markers as a useful screening tool for identifying HCM patients at increased risk of adverse outcomes (Pay et al., 2024).

Immunological profiling via ssGSEA revealed distinct immune cell infiltration patterns between HCM and control groups, particularly elevated levels of activated CD8^+^ T cells. The activation of these cells may reflect an autoimmune response against aberrant myocardial antigens, such as mutant sarcomeric proteins (Massie et al., 2025). This misdirected immune attack can induce cardiomyocyte death and fibrosis, thereby perpetuating cardiac remodeling (Garmany et al., 2023). These findings suggest that targeting inflammatory pathways may represent a novel therapeutic strategy for HCM, as highlighted by Fonfara et al. (2021).

Moreover, elevated activated CD8^+^ T cells in HCM, alongside reduced activated B cells and dendritic cells (DCs), link to clinical phenotypes including fibrosis and obstruction (Zhao et al., 2022). Activated CD8^+^ T cells, characterized by cytotoxic activity, may induce myocardial injury and fibrotic remodeling, which is a key driver of left ventricular stiffness and outflow obstruction. Reduced B cells and DCs—critical for adaptive immunity—suggest immune dysregulation, potentially impairing inflammation resolution or antigen presentation rather than indicating global immunosuppression (Tursi et al., 2025). These patterns, validated across cohorts, likely reflect a myocardial stress-induced immune response to sarcomeric dysfunction or fibrosis, though causality remains unestablished. Future studies integrating immune profiling with fibrosis severity scores or functional assays will clarify whether these signatures predict disease progression or represent targets for immunomodulatory therapies. The findings highlight a dysregulated adaptive immune landscape in HCM, warranting exploration of cell-type-specific pathways in fibrosis-immunity crosstalk.

The construction of a diagnostic model using 12 machine-learning algorithms and 10-fold cross-validation represents a significant advancement in HCM biomarker discovery. The identified 12-gene signature demonstrated robust performance in both training and external validation cohorts, outperforming previous models that relied on single-omics datasets (Sheng et al., 2025). Notably, RASD1, a regulator of G-protein signaling, has been implicated in β-adrenergic hyper-responsiveness, a hallmark of HCM (Kuang et al., 2024). Studies also found that RASD1 had important implications for the early diagnosis and treatment of HCM (Gu et al., 2024; You and Dong, 2023). The COMP gene was significantly expressed in distinct hypertrophic obstructive cardiomyopathy (HOCM) subtypes, highlighting its potential role in the molecular classification and pathogenic processes of HOCM (Qin et al., 2021). Most importantly, S100A8 and S100A9 were identified as potential biomarkers for distinguishing HCM from healthy controls, primarily expressed by infiltrating M1 proinflammatory macrophages in the cardiac immune microenvironment (Zhao et al., 2022). Their enrichment in HCM suggests a role in driving proinflammatory pathways, potentially contributing to myocardial fibrosis and immune-mediated injury—critical pathological features of HCM. In addition, SFRP4 was significantly upregulated in HCM patients, demonstrating good predictive value for HCM. Functional enrichment analysis linked SFRP4 to pathways critical for HCM pathogenesis, including extracellular matrix remodeling and fibrosis: hallmark processes in myocardial structural and functional dysfunction (Ma et al., 2021). ESM1 may contribute to HCM pathogenesis by inducing coronary vasculature developmental defects and reducing compact zone cardiomyocyte proliferation (Wang et al., 2022), potentially impairing myocardial blood supply and compensatory growth, which could lead to ischemic stress and abnormal ventricular wall thickening characteristic of HCM. A novel miR-138-5p/CA3 axis involved in the pathogenesis of cardiomyocyte hypertrophy, suggesting potential therapeutic avenues for this heart disease (Chu et al., 2025). Moreover, IL-33/IL1RL1 signaling could activate TGF-β-mediated fibroblast activation and epithelial-mesenchymal transition in the myocardium, promoting extracellular matrix (ECM) production, thereby driving myocardial fibrosis and structural remodeling characteristic of HCM (Zhu et al., 2023). In addition, the genes potentially related to myocardial cell fibrosis include MYL1 (Srivastava et al., 2024), and MCEMP1 (Perrot et al., 2024). Furthermore, MT1A contribute to metabolic regulation and oxidative stress resistance (Hassan et al., 2024), protecting cardiomyocytes from energy depletion and metal ion imbalance. VGLL2 plays a direct role in regulating mitochondrial function (Honda et al., 2024), and thus may have a potential association with the onset of mitochondrial HCM (Zhuang et al., 2023). Collectively, these genes highlight the multi-factorial nature of HCM pathogenesis, with future studies warranted to validate their roles in functional assays and clinical cohorts.

The integration of the Lasso and Stepglm[both] algorithms enhanced model interpretability by reducing overfitting, a common limitation in machine learning studies. These results validate the utility of multialgorithmic approaches in precision medicine. Previous bioinformatics studies on HCM have primarily focused on small sample sizes (Wang et al., 2023) or single-omics datasets, limiting generalizability (Qin et al., 2021). In contrast, this study utilized two independent RNA-seq datasets (GSE230585 and GSE249925), with external validation in GSE180313 further strengthening the robustness of the findings. Moreover, the inclusion of immune infiltration analysis adds a novel dimension to HCM biomarker discovery, complementing the recent work by Hou et al., which identified immune-related genes in the diagnosis and management of HCM (Hou et al., 2024).

To address gene selection stability, we have used 10-fold cross-validation, we evaluated the stability of the 12 candidate genes and found that all 12 genes are consistently included in cross-validation folds. This is attributed to the L1 regularization of the Lasso algorithm, which penalizes irrelevant genes by shrinking their coefficients to zero, ensuring that key genes remain selected across different data partitions. These analyses confirm that the 12-gene signature is not coincidental but strongly associated with HCM across multiple data resamplings, providing empirical evidence for the model’s robustness. Therefore, the 12-gene signature reported here not only improves diagnostic accuracy but also provides mechanistic insights into HCM pathogenesis, particularly regarding the interplay among fibrosis, inflammation, and calcium homeostasis.

The validation cohort, in which 48% of participants carried pathogenic sarcomeric variants (predominantly MYBPC3, MYH7) and 52% had variants of unknown significance (VUS) or no mutations, reflects the genetic heterogeneity of HCM (Ranjbarvaziri et al., 2021). The training cohort focused on obstructive HCM, the most prevalent symptomatic subtype (Saddique et al., 2025), with genetic specificity controlled by an independent MYBPC3 truncation mutation subgroup and secondary hypertrophy excluded by ruling out aortic stenosis-related left ventricular hypertrophy (LVH) (Ananthamohan et al., 2024). This design rigor—integrating diverse genetic profiles (known mutations, VUS, non-mutation cases)—mitigates bias and demonstrates the signature’s robustness across heterogeneous populations, a critical feature for translating findings into clinical utility. The inclusion of healthy controls further validates the signature’s ability to distinguish HCM, underscoring the stringency of cohort selection in isolating disease-specific molecular signals. While the current study focused on obstructive HCM, the 12-gene signature’s generalizability to non-obstructive subtypes remains untested. HCM’s phenotypic diversity, driven by genetic and anatomical variation, may lead to distinct transcriptomic profiles in non-obstructive forms. For example, apical HCM is associated with unique remodeling patterns and clinical outcomes, which could alter the expression of genes related to fibrosis (Gasior, 2024). Future studies should validate the signature in well-characterized non-obstructive and mixed HCM cohorts to assess its robustness across subtypes, ensuring clinical utility beyond the obstructive phenotype.

This study establishes a foundation for future research in HCM diagnostics and pathogenesis. However, the external validation cohort did not report detailed demographic data, which could influence transcriptomic profiles and model generalizability. Gender- and age-specific differences in HCM pathogenesis (Ji et al., 2025) or ethnic disparities in genetic variants (Kraus et al., 2024) may alter gene expression patterns. To address this, future studies should include diverse populations and systematically evaluate demographic impacts on the 12-gene signature. Additionally, integrating the signature into multi-center cohorts with mixed HCM subtypes (obstructive, non-obstructive, apical) will clarify its utility across the phenotypic spectrum. Prospective trials in familial HCM screening programs, which often include asymptomatic carriers and early-stage patients, could further assess its value in early detection. Furthermore, functional investigations, such as CRISPR-mediated gene editing in cardiomyocytes, can elucidate the role of genes like VGLL2 in HCM pathogenesis (Dutton et al., 2024). Integrating multi-omics datasets (e.g., proteomics, metabolomics) may uncover novel therapeutic targets (Wu et al., 2025). In clinical practice, incorporating the diagnostic model into risk stratification algorithms could enhance HCM diagnosis. Concrete steps include initiating collaborations with multinational clinical centers to prospectively validate the signature in large, ethnically and clinically diverse cohorts, ensuring reliability across varied patient demographics. Standardized protocols for sample collection, RNA extraction, and data analysis would be established to maintain methodological consistency. For assay development, partnerships with diagnostic technology providers could expedite the creation of high-throughput, cost-effective platforms optimized for detecting the 12-gene expression signature, facilitating its translation into clinical practice.

A researcher’s selection of algorithms can be strongly shaped by individual preferences and inherent biases (Qin et al., 2023; Liu et al., 2022). To address this, we integrated various machine learning techniques and compared their diagnostic capabilities to identify the optimal model, thereby minimizing bias stemming from such subjective factors. An integrated approach using 12 algorithms across 113 combinatorial evaluations determined that a hybrid model combining Lasso and Stepglm[both] was best suited for analyzing the 12 key genes. This strategy effectively reduced dimensionality and uncovered underlying patterns, enabling the development of a simplified, clinically translatable model. Although our model includes a larger gene set than some existing HCM models (Pavic et al., 2024; Ma et al., 2024), this increased complexity may present challenges for clinical implementation. As a result, future research should focus on creating parsimonious gene signatures that maintain predictive accuracy while comprising fewer genes. Such streamlined models would better balance precision with clinical practicality, facilitating broader adoption in real-world healthcare settings.

Simultaneously, collaboration with bioinformatics experts and electronic health record (EHR) system developers would facilitate the integration of the signature into existing risk-stratification algorithms. Clinician-friendly interfaces, coupled with continuous validation using real-world clinical feedback, would enhance the tool’s diagnostic accuracy and clinical utility. This multi-pronged approach—encompassing collaborative validation, assay optimization, and algorithmic integration—would systematically translate the 12-gene signature from research discovery into a practical clinical diagnostic tool, thereby enhancing its clinical relevance and impact. While the 12-gene signature demonstrates robust diagnostic performance in clinically diagnosed HCM cases, its utility for early detection in asymptomatic mutation carriers or early-stage patients remains unproven. Transcriptomic changes in pre-symptomatic individuals may differ from overt disease, as compensatory mechanisms could mask dysregulated pathways like fibrosis or inflammation. Prospective studies in familial HCM cohorts—where asymptomatic at-risk individuals undergo regular screening—are essential to validate the signature’s ability to identify early-stage disease. Such studies would clarify whether the signature precedes clinical symptoms, enhancing its potential for preemptive intervention.

## Limitations

Despite these advancements, several study limitations warrant consideration. First, reliance on publicly available datasets restricts analysis to transcriptomic data, precluding validation at the protein and epigenetic levels (Guo et al., 2023). Second, the external validation cohort (GSE180313) had a small sample size, necessitating replication in larger, ethnically diverse populations. Third, the diagnostic model’s clinical utility remains untested in real-world settings, and its capacity to predict disease progression or inform therapeutic decisions must be prospectively validated (Pavic et al., 2024).

Targeted experimental approaches are proposed for further analysis: (1) Immunohistochemistry (IHC) on human myocardial tissue microarrays to validate protein expression of key signature genes and correlate with transcriptomic data, enabling spatial and cellular localization; (2) Western blotting in human cardiomyocytes harboring HCM mutations to assess protein levels under stress conditions, linking transcriptional changes to functional phenotypes; (3) Enzyme-linked immunosorbent assay (ELISA)-based quantification of circulating biomarkers in patient serum to evaluate non-invasive diagnostic potential; and (4) Quantitative proteomics on matched samples to integrate protein abundance data with RNA-seq, identifying post-translational regulators for mechanistic investigations. These methods would validate findings at the protein level, resolve transcriptome-protein discordances, and prioritize candidates for functional validation, bridging the gap between transcriptional signatures and biological relevance.

Although 10-fold cross-validation and an independent external cohort (GSE180313) were employed to assess model generalizability, the high-dimensional nature of transcriptomic data inherently poses overfitting risks. A critical limitation is the lack of formal overfitting evaluations, such as learning curves and permutation tests. Without these, it is not possible to fully exclude that the observed high AUC values stem from chance correlations in the data, particularly given the small sample size in the external validation cohort. Moreover, while Lasso regularization reduced the feature space, feature importance was not explicitly quantified using methods such as permutation importance or SHapley Additive exPlanations (SHAP) values, which are essential for interpreting model reliability in high-dimensional contexts. Additionally, the study did not perform *a priori* power calculations to determine the optimal sample size for detecting differential gene expression or model performance, which may have influenced the robustness of statistical comparisons and the reliability of machine learning results. Future studies should incorporate power analyses to ensure adequate sample sizes for key objectives. Finally, the study did not account for confounding variables such as medication use or comorbidities, which may influence transcriptomic profiles.

## Conclusion

In summary, this study integrated bioinformatics and machine learning approaches to identify a novel 12-gene signature for HCM, elucidating the interplay between fibrosis, inflammation, and genetic dysregulation in HCM pathogenesis. The model’s robust diagnostic performance and the mechanistic insights derived from it mark significant advancements in HCM research. However, translational validation and functional studies remain essential to fully realize its clinical potential. Overall, these findings contribute to the growing evidence base for precision medicine in HCM, with implications for early diagnosis and targeted therapy.

## Data Availability

The original contributions presented in the study are included in the article/Supplementary Material, further inquiries can be directed to the corresponding author.
